# Dilemmas for the pathologist in the oncologic assessment of pancreatoduodenectomy specimens: An overview of different grossing approaches and the relevance of the histopathological characteristics in the oncologic assessment of pancreatoduodenectomy specimens

**DOI:** 10.1007/s00428-018-2321-5

**Published:** 2018-03-27

**Authors:** Eline Soer, Lodewijk Brosens, Marc van de Vijver, Frederike Dijk, Marie-Louise van Velthuysen, Arantza Farina-Sarasqueta, Hans Morreau, Johan Offerhaus, Lianne Koens, Joanne Verheij

**Affiliations:** 1 Department of pathology, Academic Medical Center, Meibergdreef 9, 1105 AZ Amsterdam, The Netherlands; 2 Department of pathology, University Medical Center, Utrecht, Netherlands; 3 Department of pathology, Radboud Medical Center, Nijmegen, Netherlands; 4 Department of pathology, VU University Medical Center, Amsterdam, Netherlands; 5 Department of pathology, Erasmus Medical Center, Rotterdam, Netherlands; 6 Department of pathology, Leiden Medical Center, Leiden, Netherlands

**Keywords:** Pancreaticoduodenectomy, Pancreatic ductal carcinoma, Surgical pathology, Grossing technique

## Abstract

A pancreatoduodenectomy specimen is complex, and there is much debate on how it is best approached by the pathologist. In this review, we provide an overview of topics relevant for current clinical practice in terms of gross dissection, and macro- and microscopic assessment of the pancreatoduodenectomy specimen with a suspicion of suspected pancreatic cancer. Tumor origin, tumor size, degree of differentiation, lymph node status, and resection margin status are universally accepted as prognostic for survival. However, different guidelines diverge on important issues, such as the diagnostic criteria for evaluating the completeness of resection. The macroscopic assessment of the site of origin in periampullary tumors and cystic lesions is influenced by the grossing method. Bi-sectioning of the head of the pancreas may offer an advantage in this respect, as this method allows for optimal visualization of the periampullary area. However, a head-to-head comparison of the assessment of clinically relevant parameters, using axial slicing versus bi-sectioning, is not available yet and the gold standard to compare both techniques prospectively might be subject of debate. Further studies are required to validate the various dissection protocols used for pancreatoduodenectomy specimens and their specific value in the assessment of pathological parameters relevant for prognosis.

## Introduction

Pancreatoduodenectomy (PD) specimens is are among the most complex resection specimens encountered by pathologists. PD is performed most often for oncological reasons, such as (pre)cancerous lesions of the pancreas, ampulla, duodenum, and distal bile duct. In this review, we provide an overview of topics relevant for current clinical practice, in terms of both macro- and microscopic assessment of PD specimens with a suspicion of pancreatic cancer.

Careful evaluation is imperative to properly assess tumor origin for staging and to select postoperative treatment strategies. We describe how different grossing techniques may influence the assessment, and we discuss (potentially) relevant pathological parameters in terms of postoperative treatment and prognosis in relation to the current literature, i.e., tumor origin, completeness of resection, and tumor spread.

The relevance of the different histopathological characteristics has been studied extensively. Based on a retrospective analysis of 555 patients who underwent pancreatic resection for pancreatic ductal adenocarcinoma (PDAC), Brennan et al. developed a nomogram in which they present clinical and histopathological variables relevant for survival [1]. Resection margin status, degree of differentiation, number of tumor-negative and tumor-positive lymph nodes, T-stage, and tumor size were identified as relevant histopathological parameters on Cox multivariable analysis.

## Grossing techniques

To improve the quality of pathological assessment, different proposals for standardization of gross dissection protocols and multicolor inking have been published over the past decades. In our experience, two of these protocols are most commonly used. The first is the axial sectioning method: each specimen is serially sliced perpendicular to the long axis of the duodenum over its entire craniocaudal length [2].

The second method involves bi-valving of the specimen along the pancreatic duct and the common bile duct (CBD). After bi-sectioning, the two halves can be serially sliced in three different planes: either by axial slicing, multi-valving (serial slicing along each half of the pancreas), or bread loafing (parallel to the neck of the pancreas) [3].

To the best of our knowledge, there are no prospective studies comparing the different protocols head-to-head regarding establishing tumor origin, completeness of resection, and evaluation of tumor spread. A direct comparison of the different grossing techniques regarding diagnostic assessment of relevant pathological parameters is therefore not possible in this review. There is no gold standard, although The Royal College of Pathologists (RCP) favors the axial slicing method [4].

**Fig. 1:**
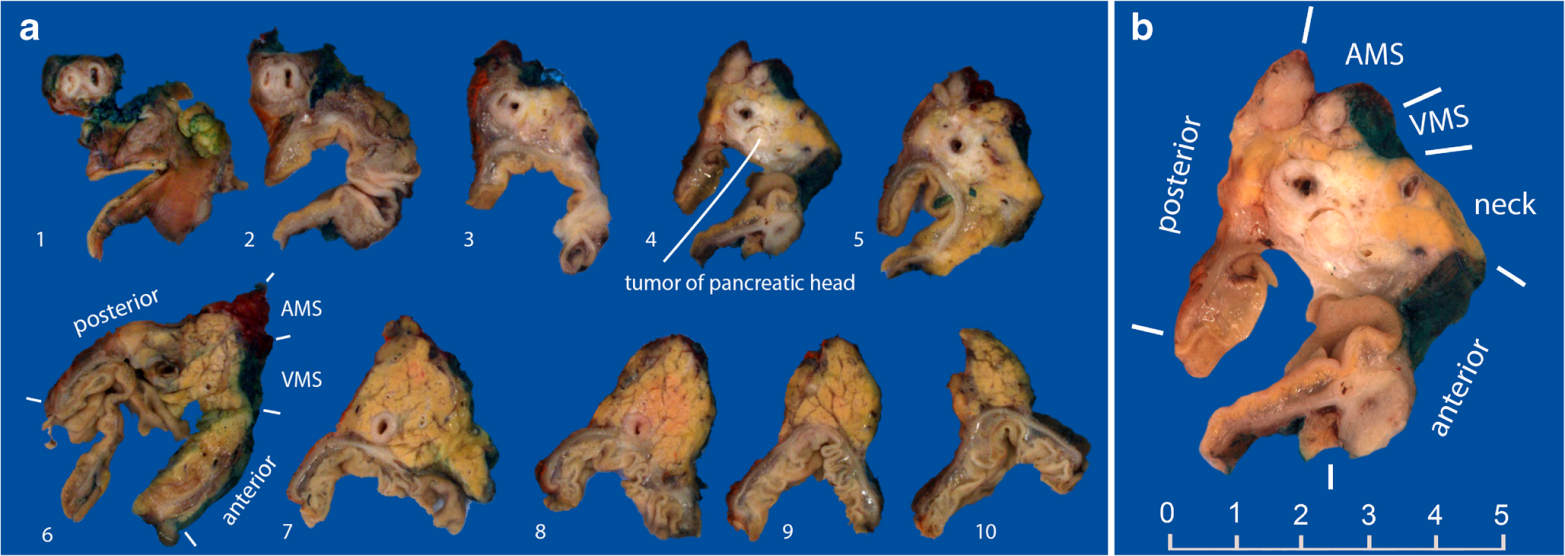
**a** Example of an axially sliced specimen. The tumor of the head of the pancreas involves the common bile duct, but does not appear to originate from it. **b** Close-up of the tumor. The margin of the neck of the pancreas has already been shaved

The axial slicing method, propagated by Verbeke, has several major advantages [2]. For every surgical specimen the same protocol is used, making it easy to perform. The circumferential margins are readily assessable (Fig.1). However, some aspects can be more difficult to evaluate using this protocol. The optimal plane of the section for capturing the ampullary region cannot be ascertained beforehand; as a result, the ampullary region frequently happens to fall between sections, hindering accurate assessment of tumor origin [3]. Moreover, the value of axial slicing in case of ampullary and cystic tumors has not been explored. This is relevant, as the origin of periampullary tumors and cystic tumors is often inconclusive on preoperative imaging. Furthermore, in case of an intraductal papillary mucinous neoplasm (IPMN), axial slicing does not allow to distinguish between lesions originating from the main pancreatic duct or side branches. Determining the precise tumor origin may be especially relevant for ampullary tumors: in a large cohort study, four subtypes of ampullary carcinomas were identified based on their origin, with differences in prognosis in this otherwise heterogeneous group of cancer [5]. This subclassification has recently been adopted by the College of American Pathologists for synoptic reporting [6].. The articles reporting on this subclassification use the bi-sectioning method for grossing. It remains to be seen whether axial slicing is suited for this subclassification of ampullary cancers [5].

**Fig. 2:**
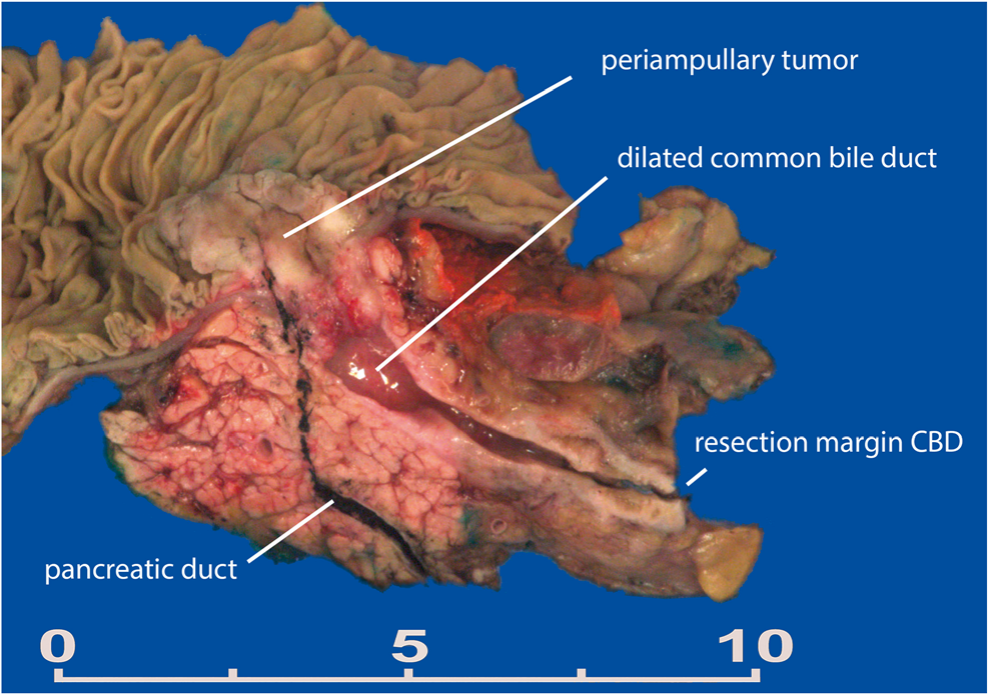
Example of a bi-valved specimen. The periampullary tumor does not involve the pancreatic or common bile duct. The common bile duct is distended due to compression by the tumor at the ampullary level

Bi-sectioning of the head of the pancreas, as described by Adsay, is technically more difficult to perform [3]. Briefly, small probes are inserted into the pancreatic duct and CBD. The head is sliced in the plane defined by the two probes, exposing both ducts longitudinally. The pancreatic duct is either approached from the ampullary orifice or from the pancreatic neck margin (Fig. 2). Probing the entire pancreatic duct is not always possible due to occlusion by tumor compression, tumor growth or reactive changes. The bi-sectioning method can be modified if needed; in case of a suspicion of distal cholangiocarcinoma, the CBD is most important to visualize, whereas the pancreatic duct takes preference in cystic lesions. However, it does have important advantages compared to axial slicing. The periampullary region is always visualized and the primary origin of periampullary tumors (pancreas, duodenum, CBD, and ampulla of Vater) can be more reliably appreciated. For ampullary tumors, the tumor origin can be more accurately assessed, which facilitates subtyping of this tumor [5]. Additionally, bi-sectioning allows much more accurate documentation of cystic tumors and their relationship to the ducts; after successful bi-sectioning, the main pancreatic duct can be completely evaluated, inked if appropriate and a distinction can be made between the CBD, main pancreatic duct, and side branches, facilitating diagnosis of main and/or side branch IPMN.

Regarding the search for lymph nodes, the RCP propagates a minimum yield of 12 lymph nodes, as this number was optimal for accurate staging of node-negative patients [4]. In order to maximize the number of harvested lymph nodes, Verbeke and Adsay describe different methods. Verbeke uses extensive perpendicular sampling of the lamellated pancreas with its surrounding soft tissue, whereas Adsay uses the so-called “orange peeling method” to procure a maximum number of lymph nodes [7]. The orange peeling method consists of shaving off the all peripancreatic soft tissue after multicolor inking. The shaved off tissue derived from the superior mesenteric vessel margin is bread-loafed and submitted entirely. Potential tumor-involved margins can also be submitted in their entirety, enabling margin assessment. Manual lymph node dissection is performed on the remaining tissue. Manual dissection may reduce double counting of lymph nodes, as the retrieved lymph nodes can be submitted in one cassette. The left-over fatty tissue is then submitted for the detection of microscopic lymph nodes. The authors conclude that the orange peeling method considerably increases the lymph node yield; however, it is unclear against which “conventional” method of searching for lymph nodes it was compared. The increased lymph node yield might be explained by the fact that more fatty tissue is included for microscopic examination. Double counting of lymph nodes remains an issue of concern in both methods.

In many institutions, a small sample of fresh tumor tissue is harvested for research purposes. To do so, multicolor inking and slicing should be performed on the freshly received specimen. The difficulty in distinguishing tumor tissue from chronic and fibrosing inflammation in the surrounding pancreatic tissue makes correct sampling challenging. A good view of the tumor is imperative. There is no literature on which method is optimal for fresh tumor sampling.

## Tumor characteristics

Site of origin, tumor size and tumor type are important parameters that should be considered when evaluating a PD specimen. Each parameter has its own challenges, and we review the parameters that should be considered when evaluating a PD specimen with a suspicion of PDAC.

### Site of origin

When PD is performed for a malignant tumor, one of the first steps is to ascertain the site of origin of the primary tumor, which cannot always be easily assessed in this anatomically complex area. The papilla of Vater is the protrusion into the duodenal lumen caused by the ampulla of Vater, which is formed by dilated junction of the distal pancreatic duct and the distal CBD. The ampulla of Vater is surrounded by the sphincter of Oddi. In the periampullary region, three distinct types of epithelial lining are joining: the duodenal surface of the ampulla, lined by intestinal epithelium; the ampulla of Vater, lined by foveolar-like mucosa with scattered goblet cells; and the distal ends of the CBD and pancreatic duct, lined by pancreatobiliary-type epithelium. Pancreatic duct glands and the peribiliary glands harbor pancreatic stem cells and biliary tree progenitor cells, and these may contribute to tumor heterogeneity [8]. Due to the complex anatomy, the periampullary area gives rise to a heterogeneous group of tumors, each with their own histologic features and biological behavior [9].

The minor duodenal papilla, which drains the accessory duct of Santorini, is situated 2 cm proximal to the major papilla. It is usually identifiable by close inspection, unless it is obliterated by tumor or severe inflammation. The minor duodenal ampulla is lined with pancreaticobiliary-type epithelium, identical to the epithelium lining the distal CBD and pancreatic duct and surrounded by a smooth muscle layer. The muscle layer is known as the sphincter of Helly, although there is some debate whether it should be considered a proper sphincter. All tumors that can occur in the pancreatic duct and the major papilla have also been reported as occurring in the minor papilla and the duct of Santorini, and awareness of the possibility of a tumor in the minor papilla might contribute to better tumor subtyping [10].

Adenocarcinomas in the periampullary region can arise from the duodenum, ampulla of Vater, distal CBD, or pancreatic duct. Importantly, different TNM staging and adjuvant therapies apply to each of these distinct tumors [9]. In addition, the primary tumor site may be an in- or exclusion criterion for clinical trials.

In practice, the primary site of origin of the tumor is mainly determined macroscopically, based on the location of the tumor bulk. In particular in voluminous tumors, the site of origin can be difficult to assess. In general, when the tumor origin cannot be determined, most pathologists choose a default “most likely” diagnosis of PDAC, as it is most frequently encountered and has a defined treatment strategy. It is generally accepted that patients with PDAC have a worse prognosis than patients with cholangiocarcinoma or ampullary carcinoma. In fact, survival of patients with PDAC may even be overestimated due to misclassified PDACs [11]. Interestingly, two retrospective analyses of 510 and 198 PDs found that the histopathological subtype of periampullary adenocarcinomas may be a better predictor of patient survival than the site of origin [12, 13]. In these studies, patients with a pancreaticobiliary subtype of ampullary or cholangiocarcinoma had a survival like that of patients with PDAC, whereas the intestinal subtype was associated with longer survival. Unfortunately, neither study described the method of gross dissection.

### Tumor definitions

PDAC is defined by the World Health Organization (WHO) as an infiltrating epithelial neoplasm with glandular (ductal) differentiation, usually demonstrating mucin production without a predominant component of any other histological subtype. An abundant desmoplastic stromal response is a typical feature [14]. The morphologic features of extrahepatic cholangiocarcinoma are very similar to those of PDAC.

Extrahepatic (distal) cholangiocarcinoma is defined by the WHO as a malignant epithelial tumor with glandular differentiation arising in the extrahepatic biliary system [14]. This includes tumors arising in the intrapancreatic part of the CBD. It is often difficult to distinguish a tumor arising in the pancreas and secondarily involving the CBD from a tumor arising in the CBD and secondarily growing into the pancreas, in part because there are few distinct morphologic features pointing to either origin. When a distal cholangiocarcinoma or PDAC involves the entire ampulla, the pathologist faces a similar dilemma. Microscopic features that may point to a bile duct origin are dysplasia within the CBD, circumferential involvement of the bile duct by invasive carcinoma, intraglandular neutrophil-rich debris, and a small tubular growth pattern [15]. The difficulty in determining the primary origin of periampullary tumors together with the lack of a clear guidance by the WHO is a source of confusion leading to a lack of conformity in diagnosis. The incidence of distal cholangiocarcinoma is likely underestimated, as in different series the estimated incidence shows a wide range in [11]. Reevaluation of patients registered with PDAC also shows frequent misclassification of distal cholangiocarcinoma [16, 17].

Ampullary carcinoma is defined by the WHO as a gland-forming malignant epithelial neoplasm, originating in the ampulla of Vater. Only carcinomas either centered on the ampulla, or circumferentially surrounding it, or completely replacing the ampulla are considered ampullary carcinomas [14]. In large tumors, for which this criterion can no longer be assessed, the presence of precursor lesions at the level of the ampulla may be of help. There is no specific subclassification for tumors arising from the different compartments of the ampulla of Vater. Owing to the lack of a clear description of what encompasses the “ampulla of Vater,” the significance of tumors arising from different sites within this complex region has not yet been elucidated. To reduce ambiguity of the entity, ampullary carcinomas are sometimes subclassified based on location into four categories, namely intra-ampullary, ampullary-ductal, periampullary duodenal, and ampullary carcinoma not otherwise specified. The categories were proposed after a retrospective analysis of 249 ampullary carcinomas, each category with a difference in survival [5]. However, this subclassification needs further validation.

### Tumor size

Tumor size (defined as the largest dimension of the tumor as assessed at pathology) is a well-established predictor of survival in PDAC and determines T-category for tumors limited to the pancreas. Generally, patients with a tumor size < 3 cm have a better prognosis, but this is mostly only significant in univariate analyses [18–20]. Multivariate analysis with correction for spread into peripancreatic soft tissue and surrounding structures is occasionally applied [21]. Saka et al. described staging based on tumor size—rather than T-category—as a viable method [22]. Multivariate analysis was used, with adjustment for age, sex, International Union Against Cancer (UICC) N-stage, margin status, and lymphovascular/perineural invasion. T-category was not considered, but more than 95% of cases had peripancreatic soft tissue involvement. Cutoff values of ≤ 2, > 2–4, and > 4 cm were found highly significant in terms of prognosis, both in their own cohort of 223 PD specimens and in the SEER database. In the eighth edition of the TNM, which came into effect in January 2018, peripancreatic soft tissue involvement is no longer a factor in the determination of T-category. T1-T3 is dependent only on tumor size, whilst T4 requires tumor involvement of the coeliac axis, superior mesenteric artery, and/or common hepatic artery [23].

### Intraductal papillary mucinous neoplasm

IPMN is a precursor lesion to PDAC that is regularly seen in clinical practice and has received much attention lately. Histologically, gastric-type, intestinal-type, oncocytic type, and pancreatobiliary-type IPMNs are discriminated. These different histological subtypes have been associated with different clinicopathological features, such as risk of high-grade dysplasia and malignant transformation. However, there is a debate about the clinical relevance of these subtypes since multiple subtypes are often present within the same IPMN and histological subtyping is difficult to reproduce in a substantial number of cases [24].

IPMNs are also subclassified as main duct-type or side branch-type, based on the location of involvement of the pancreatic duct, which is assessed by imaging. In addition, some IPMNs involve both the main pancreatic duct and the side branches and are called mixed-type IPMNs. IPMNs confined to a side branch rarely evolve into malignancy and have a better prognosis than main duct and mixed-type IPMNs [25–28]. There are no studies comparing the correlation between imaging findings and pathological findings in the distinction between main and side branch IPMN. Macroscopically, bi-valving will visualize the entire main pancreatic duct, potentially facilitating the determination of IPMN location.

PDACs arising in an IPMN have a better prognosis than PDACs not associated with IPMN. When assessing the size of a tumor arising in an IPMN, only the invasive portion should be taken into account to determine the T-category [29]. However, it is often difficult to discriminate the invasive from the non-invasive part macroscopically. In addition, multifocality can make it difficult to measure the diameter of the invasive component.

## Perineural and vasoinvasive growth

While both the presence of perineural and vasoinvasive growth have long been established as poor prognostic factors for many malignancies including PDAC, little is published about the value of these parameters in PDAC. Although some studies have shown that perineural and vasoinvasive growth are predictive of a worse outcome in univariate or multivariate analysis [30–32], other studies did not confirm this [20, 33, 34]. The incidence of perineural invasion varies between 31 and 92% across studies. The reported incidence of vascular invasion is lower, varying between 9 and 55% [20, 30–32]. As vascular elastic stains are not commonly used in the assessment of PDAC, vascular invasion can be easily missed.

## Assessment of resection margins

Multiple names are used to designate the different margins of the PD specimen. (See Table 1*).* Here, we use the names used by the RCP. The transection margins are the pancreatic neck margin, the CBD, the proximal stomach or duodenum and the distal duodenum or jejunum margin. The superior mesenteric vessel margin (including the superior mesenteric vein and artery margin) is considered a dissection margin. The superior mesenteric vessel margin is most frequently involved by tumor cells, most likely due to the lack of peripancreatic soft tissue in this area [35, 36]. The anterior surface is covered by peritoneum and considered a “free surface” rather than a dissection margin. Even so, involvement of this surface likely increases the risk of recurrence [37]. According to the RCP, the anterior surface should be considered in margin assessment. In contrast, the College of American Pathologists does not consider the free anterior surface for tumor involvement [38].

**Table 1:** Different margin names

RCP name	Also used
Superior mesenteric vessel - Superior mesenteric vein margin - Superior mesenteric artery margin	Medial margin Uncinate margin Retroperitoneal margin Inferior-posterior (retroperitoneal) margin Mesopancreatic margin Medial margin Vascular margin
Posterior margin	(part of) uncinated margin (part of) retroperitoneal margin Deep retroperitoneal posterior surface
Proximal duodenal/gastric	
Distal duodenal/jejunal	
Pancreatic neck margin	Pancreatic duct margin
Bile duct margin	
Common bile duct margin	
Anterior free surface	Anterior margin

The same discussion applies to the posterior surface. As argued by some, the posterior-right aspect of the pancreas—where the pancreatic head transitions into the duodenum—is covered by smooth connective tissue, rendering it a free margin [3]. However, many consider the posterior margin a dissection margin because the pancreas is dissected from the surrounding retroperitoneal soft tissue [39]. In colon cancer, tumor extension into the overlying peritoneum is relevant for the T-category and is associated with decreased survival [40]. Whether this is also true for pancreatic cancer has not been investigated to our knowledge.

### R1 resection

The definition of a microscopic incomplete (R1) resection differs across countries and centers. The UICC defines R1 as microscopic residual disease, without further specifying the type of margin (transection or dissection) or the mode of propagation (direct or indirect). In Europe and Japan, the presence of tumor cells < 1 mm from the resection margin is generally considered an incomplete resection, whereas in the USA, a resection is considered incomplete only when tumor cells are present in the margin. The rule of 1 mm clearance is adopted from the circumferential margin assessment in rectal carcinoma. In pancreas resection specimens, studies have shown that an R0 resection only carries any prognostic value when it is defined as ≥ 1 mm margin clearance [41, 42]. Two studies found that a margin clearance of ≥ 1.5 or ≥ 2 mm, respectively, is an even better predictor of survival than a margin clearance of ≥ 1 mm [43, 44]. However, after correction for tumor size and other clinicopathological parameters, margin clearance only remained an independent prognostic factor in the study that used a margin clearance of ≥ 1.5 mm. Recently, a prospective study evaluated the relevance of resection margin status for survival in 561 patients, of whom the majority had received adjuvant treatment [36]. Of these patients 80% had an R1 resection (< 1 mm clearance), of which 58% had direct margin involvement (0 mm margin clearance). In multivariate analysis, R1-status was independently associated with survival; a tumor clearance of ≥ 1 mm best identified the subgroup with favorable survival. The RCP pointed out that the ≥ 1 mm margin clearance should only apply to true transection and dissection margins, excluding the anterior free surface [4].

The definition which is used for microscopic incomplete resection affects tumor sampling and the number of blocks to be taken. When an incomplete resection is defined as direct involvement of the margin, the peripancreatic tissue may be sampled without special care for tissue orientation (e.g., orange peeling) [3]. However, when defining R1 as < 1 mm clearance, the relation between the inked margin and underlying (fatty) tissue must be preserved, necessitating extensive perpendicular sampling of margins that are threatened on macroscopic assessment [37]

### R1 percentage as a quality parameter

The percentage of R1 resections is often considered the most important quality parameter of pathologic assessment of PD specimens. In general, it is thought that meticulous assessment should result in an R1-percentage of 70% or higher [37, 45]. Verbeke states that more R1 resections are detected in the axial slicing method when compared to “traditional” (i.e., not axial slicing) grossing methods, and that the axial slicing method is therefore more sensitive for R1 resections [2]. For the nine studies that are considered “traditional,” the grossing method is often not clearly described. Moreover, the definition of an R1 resection is not uniform: the included margins, as well as the definition of a positive margin differ, being 0 mm in some studies and < 1 mm in others. In our opinion, further studies are needed to adequately compare the different grossing techniques in terms of R1-percentage as a quality parameter. Moreover, uniform and validated definitions for R1 need to be specified.

### Indirect tumor growth within the 1-mm margin

When tumor cells are present within 1 mm of the margin other than by direct tumor spread (i.e., by lymphangio-invasion, perineural invasion, or lymph node metastasis), it is unclear if this should be considered an incomplete resection. The RCP considers these cases to be R1-resections but offers no further explanation. In contrast, for the UICC, these cases are considered to be R0-resections, except when vessel wall invasion is present within 1 mm of the resection margin [46]. Similarly, Verbeke argues that tumor cells present by perineural spread, lymphangio-invasion or lymph node metastasis within 1 mm of the margin qualifies the resection as complete, based on the following arguments [47]. Firstly, the mode of propagation and behavior of these tumor cells is different from that of tumor cells that spread by direct invasion. Secondly, tumor cells within a lymph node are encapsulated, hence the 0-mm clearance approach seems to be appropriate. However, when tumor cells breach the lymph node capsule and infiltrate the surrounding soft tissue, the 1 mm rule becomes applicable. Thirdly, lymphovascular and perineural tumor invasion are reflective of regional spread, whilst R0 resection is commonly understood to indicate successful local clearance of tumor. Locoregional tumor recurrence because of lymph node metastasis or spread along peripheral nerves cannot be prevented by an R0 resection.

## Lymph nodes

### Tumor-positive lymph nodes

Metastasis to regional lymph nodes is independently associated with poor survival in PDAC [19, 20, 48, 49], although this has not been found in all series [50, 51]. According to the UICC, regional lymph nodes are grouped into anterior pancreatoduodenal, posterior pancreatoduodenal, inferior (including the lymph nodes around the superior mesenteric vessels), CBD, coeliac, infrapyloric, and superior and proximal mesentery lymph nodes [9]. Metastasis in non-regional LNs is defined as distant metastasis (M1). The 7th edition of the UICC staging system only considers the presence or absence of regional nodal disease. In 2015, Basturk et al. analyzed in 227 PDACs the prognostic value of the other two substaging protocols used for gastrointestinal malignancies, for which lymph node assessment had been performed according to a standard protocol. Whilst the N-category of upper gastrointestinal malignancies (N0 no metastasis, N1 metastasis to 1–2 lymph nodes, N2 metastasis to > 2 lymph nodes) performed best, they found that the N-category of the lower gastrointestinal organs (N0 no metastasis, N1 metastasis to 1–3 lymph nodes, N2 metastasis to > 3 lymph nodes) had significantly more prognostic value than that used in the 7th edition of the UICC [52]. In the eighth edition of the TNM, the N-category matches that of the lower gastrointestinal organs [23]. The Japan Pancreas Society distinguishes three N-categories and gives a weighting factor according the location of the lymph nodes [53]. In published series, microscopically tumor-positive lymph nodes are found in up to 80% of surgical specimens during routine examination [48, 54].

Tumor involvement of distant lymph nodes is associated with decreased survival in pancreatic cancer patients [55]. However, extended lymphadenectomy is discouraged and seldom performed, as it has been shown to be of limited value in long term survival, whilst increasing morbidity [56].

### Direct invasion of lymph nodes

Direct invasion of lymph nodes by continuous tumor growth is present in about 5–10% of PD specimens. Two articles report no difference in survival for patients with direct nodal invasion versus those with lymph node metastasis, warranting an interpretation of direct invasion as “regular” lymph node positivity. Another article found that patients with isolated lymph node involvement did have improved survival compared to patients with metastasis to regional lymph nodes [57]. The biological mechanisms responsible for eventual differences between different modalities of tumor spread remain unclear [57, 58].

### Nodal micrometastases

A few studies have evaluated the implication of the presence of isolated tumor cells or micrometastases in lymph nodes [59–61]. The presence of nodal micrometastases identified on immunohistochemistry appears to be an independent prognostic factor for patients that were considered node-negative on routine histological examination. These patients have similar survival curves as N1 patients on routine examination. Patients with tumor negative lymph nodes by immunohistochemistry have a markedly better survival compared to patients with micrometastases. These studies did however not differentiate between nodal micrometastases and isolated tumor cells.

### Lymph node ratio

The lymph node ratio considers both the total number of lymph nodes and the number of positive lymph nodes. It has proven to be an important predictor for survival in pancreatic cancer, although the predictive value of this parameter remains proportional to the adequacy of lymph node yield [48]. In addition, it has proven more predictive than the dichotomous presence or absence of nodal disease [62].

### Extra-nodal lymph node spread

Extra-nodal tumor spread from tumor positive lymph nodes is an adverse prognostic factor in many tumor types, including rectal, thyroid, bladder and gastric cancer. Luchini et al. recently published a review on the significance of extra-nodal spread in PDAC [63]. It was associated with a poor prognosis in terms of overall and disease-specific survival. For this reason, they argue that its presence should be considered in oncologic staging and the choice of therapeutic approach. They also concluded that extra-nodal tumor spread may be present in more than 50% of N1 patients.

### Peripancreatic soft tissue spread

Spread of a tumor outside of the pancreas into the surrounding soft tissue or adjacent organs was a feature of T3 tumors according to theTNM 7 classification. The assessment of tumor spread into the peripancreatic soft tissue has a large margin of error, as the pancreas lacks a true capsule, meaning there is no clear demarcation between pancreatic and peripancreatic tissue. Peripancreatic soft tissue involvement appears to be present in most cases. Saka et al. showed that, when using the orange peeling method for the peripancreatic soft tissue, tumor invasion is nearly always present (> 95%) and therefore not a good predictor of survival [22]. Another complicating factor when assessing tumor spread into peripancreatic soft tissue is atrophy and fatty degeneration of the exocrine pancreas. Islet cells may be of guidance in these cases, as their presence indicates the previous level of the exocrine pancreas. In the eighth edition of the TNM, peripancreatic soft tissue spread is no longer relevant for the T-category; only tumor size is considered in the definition of T1-T3.

## Neoadjuvant treatment

An increasing proportion of patients receives neoadjuvant therapy, including chemotherapy and radiation therapy. The influence of these treatments on the clinical relevance and definition of R-status is still unknown [2, 64]. Reactive changes such as fibrosis induced by neoadjuvant therapy, in post-treatment resection specimens might complicate the macroscopic identification of the tumor. Microscopic assessment of these cases includes evaluation of the response to preoperative treatment; however, there is no consensus on how tumor regression should be graded [65]. In a large retrospective study, Chatterjee compared 233 patients with no and minimal residual disease to patients with moderate and poor response [66]. They found that patients with no or minimal residual disease have significantly improved survival. This advantage remained after multivariate analysis including pathologic tumor stage, margin status, and lymph node status. They conclude that histologic grading is an important prognostic factor. Several grading schemes for the assessment of residual tumor in post-treatment PD specimens have been proposed, including the College of American Pathologists (CAP) and Evans grading system [67, 68]. Recently, Lee et al. validated their own 3-tiered histologic tumor regression grading (HTGR) scheme (HTRG 0, no viable tumor; HTRG 1, < 5% viable tumor cells; HTRG 2, ≥ 5% viable tumor cells). In multivariate analysis, HTRG grade 0 or 1 was an independent prognostic factor for better disease-free survival, but not for overall survival [69]. In a recent retrospective cohort study, 398 PDAC patients who underwent neoadjuvant therapy and PD were analyzed to validate the new size-based T-category definitions of the eight edition of the TNM [70]. The authors showed that the new T stage system outperformed the old T stage system in patients after neoadjuvant treatment. Additionally, they found that a tumor size cutoff of 1.0 cm worked better for T2 than the proposed tumor size cutoff of 2.0 cm in this group of patients. Interestingly, all nine patients with a complete pathologic response showed no recurrence at the end of follow-up.

## Conclusion

We presented a comprehensive overview of the dilemmas the pathologist may face in the assessment of a PD specimen. Several different approached for gross dissection have been proposed in the literature, each having its advantages and disadvantages about assessment of important clinicopathological parameters. Compared with axial slicing, bi-valving seems better suited for the assessment of tumor origin. The value in terms of prognosis of some the clinicopathological parameters (e.g., tumor size) has been evaluated by many studies. However, most of these studies were retrospective and the protocols and frequently used definitions lacked a standardized histopathological approach, both at the macroscopic and microscopic level. For several pathological parameters, e.g., completeness of resection, this has hampered clinical validation and further application. As a result, different organizations have published their own guidelines, which show divergence on potentially important issues. Pathologists and surgeons should be aware of these differences and of the uncertainties in histopathological assessment of PDs. Neoadjuvant therapy, which is increasingly administered, will also influence the assessment of the specimen and the interpretation of certain parameters in the resection specimen after chemotherapy [65]. Further prospective studies are needed to validate the clinical relevance of the various dissection protocols and the interpretation of certain macroscopic and microscopic parameters.
